# Individual‐based analysis of hair corticosterone reveals factors influencing chronic stress in the American pika

**DOI:** 10.1002/ece3.3009

**Published:** 2017-04-26

**Authors:** Matthew D. Waterhouse, Bryson Sjodin, Chris Ray, Liesl Erb, Jennifer Wilkening, Michael A. Russello

**Affiliations:** ^1^ Department of BiologyUniversity of British ColumbiaKelownaBCCanada; ^2^ Institute of Arctic and Alpine Research and Department of Ecology and Evolutionary BiologyUniversity of ColoradoBoulderCOUSA; ^3^ Departments of Biology and Environmental StudiesWarren Wilson CollegeAshevilleNCUSA

**Keywords:** body mass, climate change, glucocorticoids, mammal, metabolic rate, thermal stress, wildlife

## Abstract

Glucocorticoids are often measured in wildlife to assess physiological responses to environmental or ecological stress. Hair, blood, saliva, or fecal samples are generally used depending on the timescale of the stress response being investigated and species‐specific considerations. Here, we report the first use of hair samples to measure long‐term corticosterone levels in the climate‐sensitive American pika (*Ochotona princeps*). We validated an immunoassay‐based measurement of corticosterone extracted from hair samples and compared corticosterone estimates obtained from plasma, hair, and fecal samples of nine pikas. To demonstrate an ecological application of this technique, we characterized physiological stress in 49 pikas sampled and released at eight sites along two elevational transects. Microclimate variation was measured at each site using both ambient and subsurface temperature sensors. We used an information theoretic approach to compare support for linear, mixed‐effects models relating corticosterone estimates to microclimate, body size, and sex. Corticosterone was measured accurately in pika hair samples after correcting for the influence of sample mass on corticosterone extraction efficiency. Hair‐ and plasma‐based estimates of corticosterone were weakly correlated. The best‐supported model suggested that corticosterone was lower in larger, male pikas, and at locations with higher ambient temperatures in summer. Our results are consistent with a general negative relationship between body mass and glucocorticoid concentration observed across mammalian species, attributed to the higher mass‐specific metabolic rates of smaller bodied animals. The higher corticosterone levels in female pikas likely reflected the physiological demands of reproduction, as observed in a wide array of mammalian species. Additionally, we establish the first direct physiological evidence for thermal stress in the American pika through nonlethal sampling of corticosterone. Interestingly, our data suggest evidence for cold stress likely induced during the summer molting period. This technique should provide a useful tool to researchers wishing to assess chronic stress in climate‐sensitive mammals.

## 1 Introduction

Rapid environmental change represents a potential stressor and selective force on wildlife populations (Reeder & Kramer, 2005; Wingfield, Romero, & Goodman, 2001). The main physiological response to long‐term environmental stress is the activation of the hypothalamic–pituitary–adrenal axis (HPA) resulting in the release of glucocorticoids (GC), in the form of corticosterone or cortisol, into the bloodstream (Sapolsky, Romero, & Munck, 2000; Sheriff, Dantzer, Delehanty, Palme, & Boonstra, 2011). This increase in GC facilitates a suite of adaptive responses to stressful stimuli, such as behavioral changes and energy mobilization via gluconeogenesis, which can enhance short‐term survival (Wingfield, Romero, & Goodman, 2001). However, long‐term activation of the HPA signifies chronic stress and can have detrimental physiological consequences including: suppressed immune response and growth, severe protein loss, fat deposition and hypertension, as well as undesirable behavioral changes including decreased cognitive functioning, inhibition of reproductive behavior, and depression (Sapolsky et al., 2000; Wingfield et al., 1998). For these reasons, the relative levels of GC often reflect overall health and fitness (Blas, Bortolotti, Tella, Baos, & Marchant, 2007; Bonier, Martin, Moore, & Wingfield, 2009), and measurement of GC is increasingly incorporated into ecological and conservation studies (Busch & Hayward, 2009). The relative strengths and feasibility of methodologies to assess stress in wildlife has therefore been a major recent focus (reviewed in Sheriff et al., 2011).

Several techniques have been developed for measuring stress in wildlife, including measuring GC levels in hair, blood, or saliva, and measuring glucocorticoid metabolites (GCM) in fecal samples. Levels of GC in both saliva and blood respond rapidly to stress and therefore require capture techniques that allow sampling before the activation of the HPA in response to capture stress (generally 2–5 min; Sheriff et al., 2011). Where rapid sampling has been possible, this technique has revealed fundamental insights into factors governing GC levels within and across mammalian species. For instance, in a recent meta‐analysis, Haase, Long, and Gillooly (2016) found a surprisingly strong connection between plasma cortisol levels and mass‐specific metabolic rate across a wide variety of taxa, providing a predictive framework for GC levels within and among species. However, obtaining timely blood samples may not be feasible for all species. In such cases, measuring fecal GCM offers a less invasive technique for assessing stress in wildlife (Touma & Palme, 2005). Fecal samples accumulate the metabolic byproducts of stress hormones only during gut passage and therefore primarily reflect chronic stress experienced over a number of hours or days. Additionally, most species exhibit diurnal and seasonal shifts in GC (Reeder & Kramer, 2005; Sheriff et al., 2012), making it necessary to obtain a time series of samples to effectively assess long‐term chronic stress using blood, saliva, or fecal samples.

The measurement of GC incorporated into hair is a relatively new approach to assess stress in wildlife (Koren et al., 2002). While the direct mechanism by which GC is incorporated into hair is still unknown (Gow, Thomson, Rieder, Van Uum, & Koren, 2010), this sample source offers the potential to measure relative levels of GC over the duration of time the hair was grown, which typically encompasses several weeks or months. This longer‐term record makes hair analysis a powerful approach for assessing long‐term chronic stress (Russell, Koren, Rieder, & Van Uum, 2012). Despite this major advantage, only a limited number of wildlife studies have utilized hair as opposed to more established alternatives (Sheriff et al., 2011). While it is likely that hair stress analysis could provide deeper insights into climate‐induced stress in wildlife, another study cautioned that this approach may be more appropriate for detecting population rather than individual stress responses (Mastromonaco, Gunn, & Edwards, 2014). This suggestion came after analyzing long‐term trends in fecal GCM and hair GC in eastern chipmunks; there was a significant increase in GCM associated with logging but no change in hair GC. The authors concluded the time period measured by hair samples was too long to reflect individual differences in stress. However, this critique would be dependent on the research objective and species examined. If this methodology can detect individual responses to long‐term chronic stress, then it could afford important insights into physiological responses to environmental stressors in climate‐sensitive species.

**Figure 1:**
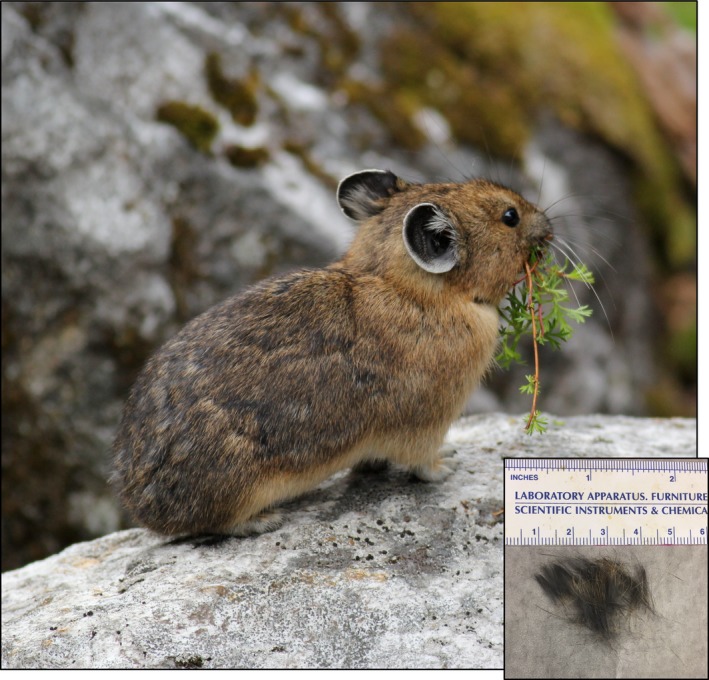
Image of an American pika and reference hair sample (inset) weighing approximately 10 mg. Photograph courtesy of Andrew Veale

The American pika, *Ochotona princeps*, is a small lagomorph generally considered to be a thermally sensitive, cold‐adapted specialist (Figure 1; MacArthur & Wang, 1974; Smith, 1974). Pikas have an exceptionally high metabolic rate (Lovegrove, 2003) and low thermal conductance (MacArthur & Wang, 1973), which allows them to survive in an alpine climate without hibernating. However, these features also result in the pika having a resting body temperature only a few degrees below its lethal threshold (MacArthur & Wang, 1974; Smith, 1974). Pikas require access to cool microclimates to behaviorally thermoregulate (Hafner, 1993; MacArthur & Wang, 1974). It is thought that this thermal sensitivity may predispose the American pika to the negative ramifications of climate change, casting them as a sentinel species for detecting the ecological consequences of climate change (Beever, Brussard, & Berger, 2003; Jeffress, Rodhouse, Ray, Wolff, & Epps, 2013; Schwalm et al., 2016; Wilkening, Ray, Beever, & Brussard, 2011). Recent analysis of pika populations in the Great Basin supports this view; the minimum elevation inhabited by pikas in the region has risen by 150 m in the past century (Grayson, 2005), and climate has been implicated in local extirpations (Beever, Ray, Wilkening, Brussard, & Mote, 2011). Therefore, assessing the biotic response to rapid environmental change in the American pika has become increasingly important as an early warning sign.

Here, we evaluated the utility of hair samples for measuring long‐term chronic stress in the American pika. First, we demonstrate the sensitivity of the assay protocol through validation, and we compare estimates of GC from hair with estimates of plasma GC and fecal GCM from the same individuals. Next, we apply this method to investigate relationships between hair GC, microclimate, body size, and sex over two elevational gradients to assess whether hair samples can provide direct insights related to climate‐mediated stress responses.

## 2 Materials and Methods

### 2.1 Sample site and sample collection

**Figure 2:**
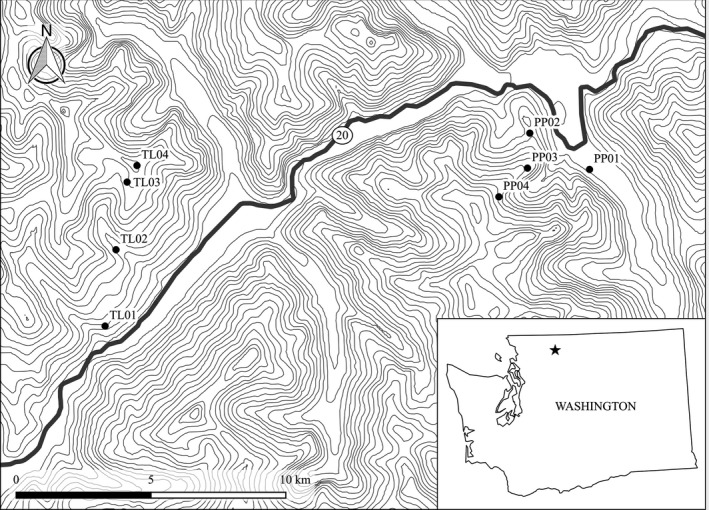
Sample sites in North Cascades National Park, Washington, USA. Thornton Lake (TL) and Pyramid Peak (PP) sampling sites shown as circles. Inset map shows approximate location in Washington state. Topographic lines represent 100‐m intervals of elevation

Sample sites were located along two previously established transects representing elevational gradients in North Cascades National Park, WA (NOCA; Russello, Waterhouse, Etter, & Johnson, 2015). Four sites were located along each transect, spanning ~1,000 m (Figure 2, Table 1). The two transects, Thornton Lakes (TL) and Pyramid Peak (PP), ran roughly southwest and northeast, respectively. Each transect included the highest (subalpine) and lowest occupied sites identified in its respective region.

**Table 1:** Site description of the Pyramid Peak (PP) and Thornton Lakes (TL) sampling transects in North Cascades National Park, WA

Site	Elevation	*Amb_max*	*Amb_day*	*Tal_max*	*Tal_day*	*Tal_night*	*n*	Cranial	Weight	*M/F*
PP01	480	25.2	22.8	16.6	16.2	15.8	5	24.5	153.0	0.67
PP02	810	23.8	20.3	14.2	13.7	13.7	6	25.6	138.3	1.00
PP03	1327	20.0	17.7	14.7	13.9	12.6	3	23.5	123.3	0.50
PP04	1550	17.8	15.6	13.1	12.4	11.4	9	23.9	152.2	2.00
TL01	504	23.6	21.2	19.2	18.3	17.9	7	27.7	182.1	0.75
TL02	760	23.3	20.6	16.3	15.8	14.6	8	29.2	182.5	1.67
TL03	1409	17.6	15.7	11.0	10.4	10.4	7	26.9	162.6	1.33
TL04	1665	15.4	14.2	12.4	11.6	10.9	4	23.8	131.3	1.00

Mean daily temperatures for ambient maximum (*Amb_max*), ambient daytime (*Amb_day*), talus maximum (*Tal_max*), talus daytime (*Tal_day*), and talus nighttime temperatures (*Tal_night*) are reported in °C along with elevation (m), sample size (*n*), mean cranial diameter (mm), mean weight (g), and sex ratio (*M/F*) for each site.

Transects were sampled between July 20 and August 29 of 2014; pikas were live‐trapped using Tomahawk (Hazelhurst, WI) model 202 collapsible traps following University of British Columbia Animal Care Protocol # A11‐0371‐006 and U.S. National Park Service Permit # NOCA‐2014‐SCI‐0022. Trapping was generally conducted between 0700 and 1100 or 1600 and 2000 when temperatures were between 5 and 18°C. After capture, each animal was transferred to a handling bag. A small tuft of fur (about 5 mm^2^; Figure 1, inset) was plucked from a hind limb, as shaving was not practical in this species. Hair samples were stored in individually labeled coin envelopes within a container of silica desiccant. Two small samples of ear tissue (3 mm in diameter) were collected and stored in ethanol for molecular sexing. Cranial diameter was measured with digital calipers to the nearest millimeter, and body mass (minus handling bag weight) was measured to the nearest 5 g using a Pesola scale.

### 2.2 Microclimate measurements

Microclimate measurements were taken at each site by deploying four temperature sensors (DS1921G Thermochron i‐Button, Maxim Integrated Products, Sunnyvale, CA, USA). Following sampling, sensors were deployed in weather‐proof housing; two “ambient sensors” were placed 1.5 m above the talus, each under a white plastic shade in neighboring trees, while two “talus sensors” were deployed in a central location at each site approximately 0.8 m below the talus surface. Temperature was recorded every four hours (starting at 02:00) during 24–31 August 2014 and June 1 to August 15 2015. To represent summer microclimatic differences among sites, mean daily maximum and mean daytime (10:00 to 18:00) temperatures were averaged for the two talus and the two ambient sensors at each site. Additionally, mean nighttime (18:00 to 10:00) talus temperatures were calculated for each site.

### 2.3 Molecular sexing

Morphological differences between male and female genitalia are poorly defined in pika (Duke, 1951); therefore, sex was determined using the molecular protocol described by Lamb, Robson, and Russello (2013). DNA was extracted from tissue samples using the Macherey‐Nagel NucleoSpin Tissue kit (Macherey‐Nagel GmbH & Co. KG, Duren, Germany) following the manufacturer's protocols. Sex was determined by the selective co‐amplification of an allosomal‐linked locus (SRY) and an autosomal control locus (Ocp 10). Scoring was conducted by running the PCR product on a 1.5% agarose gel containing 2.5% SYBR Safe (Invitrogen, Carlsbad, CA, USA). To ensure accuracy, 50% of the samples were sexed independently a second time and assigned sexes were compared.

### 2.4 Extraction of corticosterone from hair and immunoassay

Corticosterone extraction from hair samples followed Meyer, Novak, Hamel, and Rosenberg (2014) using the DetectX^®^ Corticosterone Enzyme Immunoassay (EIA) kit (Arbor Assays Design, Inc., catalog no. K014‐H1). All hair follicles were removed with a razor blade to avoid the addition of skin tissue (Gow et al., 2010). The remaining hair was added to a 15 ml tube, washed twice with 3 ml 99.7% high‐performance liquid chromatography (HPLC) grade isopropanol by rotating for 3 min, then decanted to remove external contaminants, and dried under a fume hood. Dried samples were weighed to the nearest 0.1 mg and transferred to a reinforced 2.0 ml tube with three 3.2‐mm chrome‐steel beads. Samples were pulverized in 3‐min intervals for 3–18 min at 30 Hz on a MM301 Mixer Mill (Retsch^®^, Newtown, PA). Once samples were uniformly pulverized, 1.5 ml of HPLC‐grade methanol was added to each sample and rotated for 24 hr at room temperature. Samples were then centrifuged at 13,800 g for 10 min and 1 ml of the supernatant was transferred to a 1.5‐ml microcentrifuge tube without disturbing the hair pellet. This extract was dried under a gentle stream of air in a fume hood for approximately 1–3 days until all methanol had evaporated. The extract was reconstituted using 200 μl of the EIA buffer supplied with the kit, vortexed vigorously, and then immediately frozen at −20°C until analyzed.

Each sample was run in duplicate along with six standard concentrations of corticosterone and two nonspecific binding (NSB) and two maximum binding (B_O_) wells for each plate. Absorption values at 450 nm were recorded using a Syngery HT microplate reader (Biotek, Winooski, VT, USA). Final concentrations of GC were expressed as picogram (pg) of corticosterone per milligram (mg) of washed, dried hair.

Methods used to measure hair corticosterone concentrations were validated by: (1) demonstrating parallelism between the standard curve and serial dilutions of hair extract; and (2) determining the recoverability of exogenous corticosterone added to hair extracts prior to analysis. For the addition of exogenous corticosterone, six samples were diluted 1:1 each with one of the standard curve solutions. Extraction efficiency likely varies with initial sample mass yielding proportionally higher estimates of glucocorticoids in smaller samples (Millspaugh & Washburn, 2004; Tempel & Gutierrez, 2004). All corticosterone estimates were plotted against sample mass to identify potential relationships, and, if present, a nonlinear model was fitted using the *nls* function and used to account for the influence of extraction efficiency. For further comparison, nine additional hair samples were obtained from pikas previously analyzed for plasma corticosterone (Wilkening & Ray, 2016) and baseline fecal GCM (i.e., stress levels before capture; Wilkening, Ray, & Sweazea, 2013). Plasma samples were not collected within 3 min of capture and thus measured an acute stress response, while fecal samples were collected prior to the stress signature documented for pikas (GCM increases 11–15 hr after capture, Wilkening et al., 2013) and represented a chronic stress response. Relationships between hair, plasma, and fecal GCM were assessed with a linear regression using the *lm* function. All analyses (unless otherwise noted) were conducted using the *stats* package in R version 3.1.3 (R Core Team 2015).

### 2.5 Data analysis

The distribution of each independent variable was assessed for normality according to the Shapiro Wilk test using the *shapiro.test* function, and by inspecting a normal probability plot using the *qqnorm* function. Any variable that deviated from normality was transformed using a natural log transformation and retested for normality. As elevation can have an overriding influence on most environmental parameters, we next tested for collinearity among all independent variables using the Pearson correlation coefficient and *corr.test* function in R. To reduce collinearity, where significant (α = 0.05) and strong (|*r*| ≥ 0.80) correlations were found, the more physiologically relevant variable was retained based on previous studies of pika ecology (see section 3). Mixed‐effects models were used to compare corticosterone estimates to all combinations of remaining independent variables using the *lmer* function of the *lme4* package (Bates, Maechler, Bolker, & Walker, 2014), after setting REML = ALSE to allow for model selection via AIC_*c*_. Model fit was assessed by calculating the marginal *R* ^2^ as suggested by Nakagawa and Schielzeth (2013) using the *sem.model.fits* function of the *piecewiseSEM* package (Lefcheck, 2015). The top model was tested for all the basic assumptions of linear regression including: linearity, homoscedasticity, and normality of residuals. Additionally, excessively influential data points were assessed using the *influence.ME* package (Nieuwenhuis, Te Grotenhuis, & Pelzer, 2012).

## 3 Results

### 3.1 Laboratory validation

**Figure 3:**
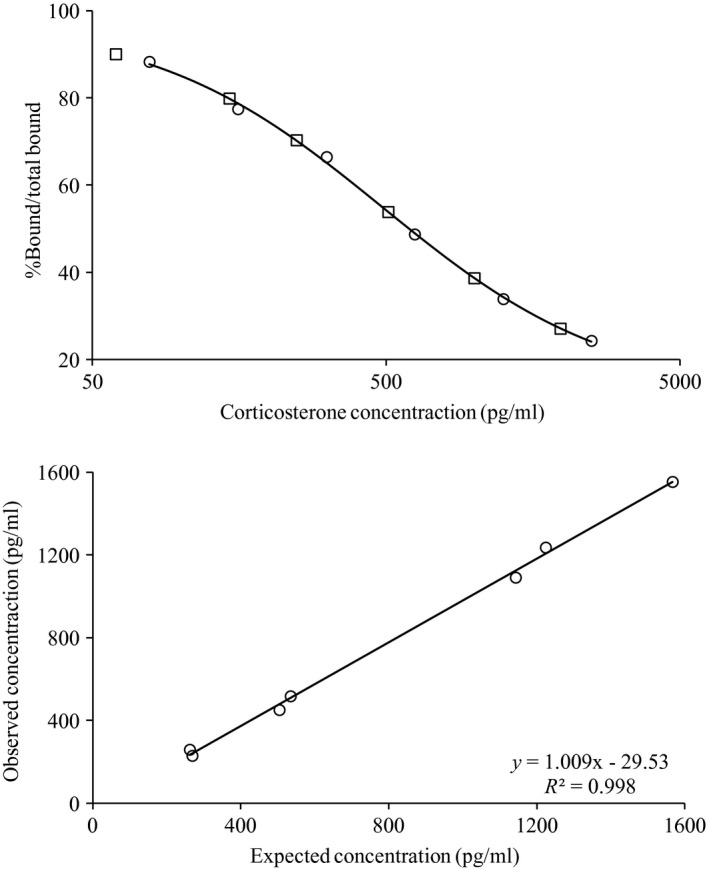
Top: Parallelism between the standard curve (solid line with circles) and serial dilutions of one sample (squares, no line). Bottom: Addition curve showing a linear relationship (*p* < .001) between observed and expected GC when samples were mixed 1:1 with standard concentrations of corticosterone from the standard curve

**Figure 4:**
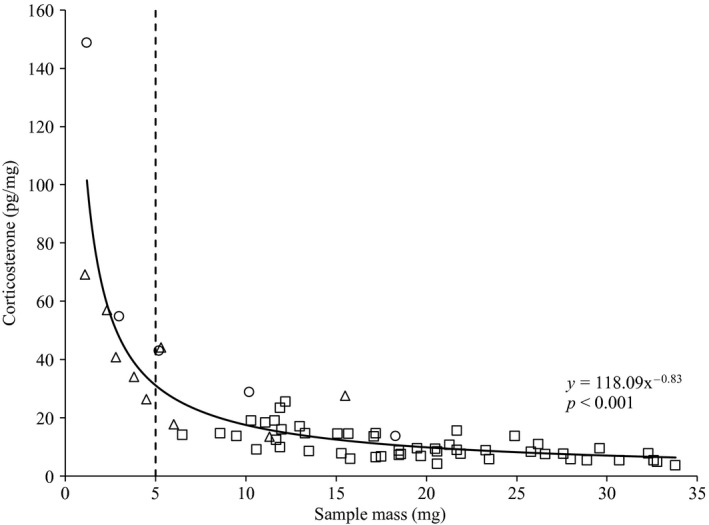
Relationship between sample mass and estimated corticosterone concentration using NOCA samples (squares), hair samples from paired plasma and fecal samples (triangles), and differing masses from PP04T08 (circles). Inset exponential relationship (solid line) was developed using all samples. Dashed line shows our suggested 5‐mg minimum sample weight cutoff

Serial dilutions showed a parallel response of samples across the entire standard curve, but the GC concentration from the highest dilution (1:24) was lower than expected relative to the standard curve (Figure 3). Due to the lower than expected GC concentrations, all remaining samples were analyzed undiluted. The addition curve showed that 99.8% of exogenous corticosterone was recoverable in the sample matrix. The mean intra‐assay variation was 2.01% and 1.88%, and the inter‐assay variation was 2.02% and 4.58% for the B_O_ and NSB standards, respectively. There was a strong nonlinear relationship between sample mass and corticosterone concentration, indicating a decrease in extraction efficiency with increasing sample mass (Figure 4). To verify this trend, varying initial quantities of hair (1.2, 3, 5.2, 10.2, and 18.3 mg) were extracted and analyzed from one sample (PP04T08) resulting in the same nonlinear relationship. A power model was fitted to the data, and all subsequent analyses were conducted on the residuals from this model to correct for extraction efficiency. The residuals of this model showed heteroscedasticity indicating sample masses less than approximately 2.3 mg could lead to inaccurate estimates of corticosterone.

### 3.2 Equivalence of corticosterone estimates from different sample sources

The sample masses for the nine hair samples used to contrast hair GC, plasma GC, and fecal GCM were low (5.8 mg ± 4.7 *SD*), and the mean coefficient of variation (CV) of corticosterone estimates was high (29.85%) between replicates. There was a substantial skew in plasma GC estimates; to facilitate comparison, plasma GC estimates were natural log transformed. There was a marginally nonsignificant positive relationship between hair and plasma GC (*F* = 3.57, *df *= 7, *R* ^2^ = .338, *p* = .101) and no relationship between hair GC and fecal GCM (F = 1.09, *df* = 7, *R* ^2^ = .134, *p* = .332). Additionally, there was no relationship between plasma GC and fecal GCM (F = 0.830, *df* = 7, *R* ^2^ = .106, *p* = .393).

### 3.3 Pika stress analysis along elevational gradients

**Figure 5:**
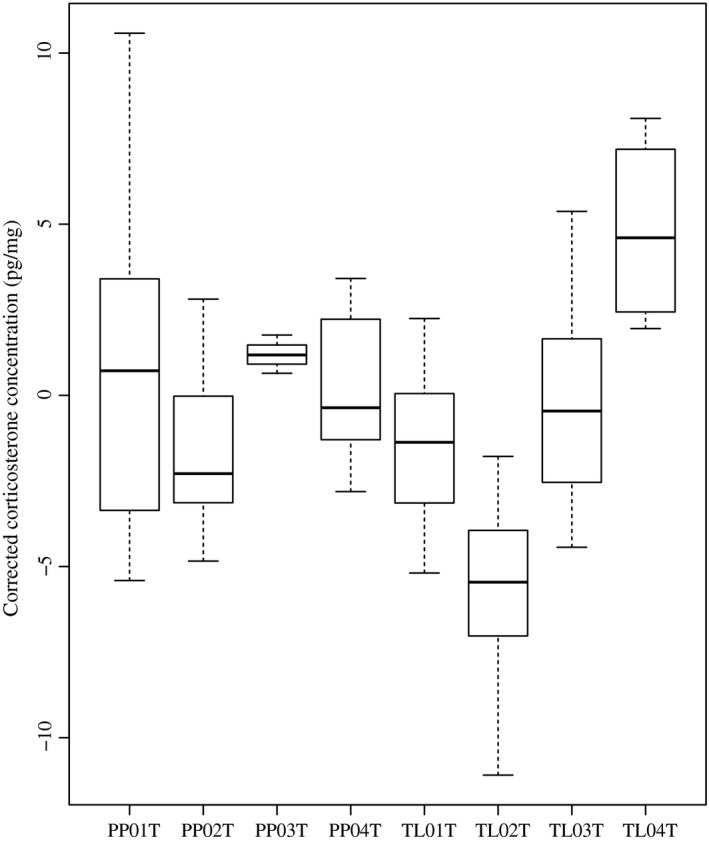
Box and whisker plot showing average corticosterone per site after correcting for extraction efficiency (see Table 1 and Figure 2 for site descriptions). Boxes represent medians, 25% and 75% quartiles while whiskers extend through 95% interquartile range. A one‐way ANOVA showed significant deviation among sites (*F* = 5.028, *df* = 7, *p* < .001). Sites are numbered to reflect relative elevation, where 01 = lowest

Hair samples were obtained from a total of 49 pikas (23 females and 26 males; Table 1). All pikas were unambiguously sexed with no replicate returning a different sex. A mean of 19.4 mg (±7.3 *SD*) of washed, trimmed hair was obtained from each sample, and the minimum sample weight was 6.5 mg. Resulting corticosterone estimates had a mean CV of 7.19% between replicates. A one‐way ANOVA showed significant deviation in corticosterone levels among the sample sites (Figure 5).

Only cranial diameter and body mass were non‐normally distributed (respectively: W = 0.948, *p* = .032; W = 0.911, *p* = .001). A natural log transformation did not establish normality nor approximate a normal distribution; therefore, nontransformed data were used in subsequent analysis. Due to significant collinearity among all temperature metrics and elevation (Table 2), elevation was eliminated in favor of a more direct assessment of microclimate variation. Similarly, mean ambient daily temperature was eliminated in favor of mean maximum daily temperature (*Amb_max*), which may be a better metric of thermal stress (Beever et al., 2011). All talus temperature metrics were collinear with *Amb_max*; however, we included *Tal_night* as this metric represented the mean nighttime temperature pika were likely subjected to, in contrast to *Amb_max*, which represented the mean maximum daytime temperature. Finally, body mass was eliminated in favor of cranial diameter as body mass is likely to fluctuate on a seasonal basis and cranial diameter was more accurately measured in the field (personal observation).

**Table 2:** Correlation matrix of independent variables (see Table 1 for definitions)

	Cranial	Weight	Elevation	*Amb_max*	*Amb_day*	*Tal_max*	*Tal_day*
Weight	0.78^a^						
Elevation	−0.35	−0.23					
*Amb_max*	0.29	0.17	−0.97^a^				
*Amb_day*	0.29	0.19	−0.98^a^	0.99^a^			
*Tal_max*	0.28	0.24	−0.86^a^	0.80^a^	0.84^a^		
*Tal_day*	0.29	0.24	−0.88^a^	0.83^a^	0.86^a^	1.00^a^	
*Tal_night*	0.30	0.24	−0.93^a^	0.86^a^	0.89^a^	0.98^a^	0.98^a^

a Indicates significance after sequential Bonferroni correction (*p* ≤ .007).

A total of 13 mixed‐effects models were assessed using *Amb_max*,* Tal_night*, sex, and cranial diameter as independent variables, including all possible models except those based on the highly collinear variables *Amb_max* and *Tal_night*. The top model incorporated all of these variables except *Tal_night* (Table 3), with lower corticosterone estimates at sites with higher ambient temperatures and for larger, male pika. The marginal *R* ^2^ for this model showed these variables explained about 36.8% of the variation in corticosterone estimates. The residuals of this model were normally distributed (W = 0.9628, *p* = .1231), showed no signs of heteroscedasticity, nor a linear relationship with the fitted value (F = 0.251, *df* = 47, *R* ^2^ = .005, *p* = .617) or with the main predictor (cranial estimates; F = 1.411E−26, *df* = 47, *R* ^2^ = 3.00E−28, *p* = 1). No excessively influential data points were identified (Cook's distance <0.85 for all sites and < 0.5 for all samples).

**Table 3:** Information theoretic analysis of mixed‐effects models explaining variation in corticosterone estimates of American pika samples (see Table 1 for definitions)

Model	K	AICc	Weight	Evidence ratio	Marinal *R* ^2^
*Cranial—Sex—Amb_max*	6	264.60	0.248	1.00	.368
*Amb_max—Cranial*	5	264.80	0.225	1.10	.326
*Cranial*	4	265.31	0.174	1.43	.208
*Sex—Cranial*	5	265.60	0.151	1.64	.239
*Tal_night—Cranial*	5	266.80	0.083	3.00	.261
*Cranial—Sex—Tal_night*	6	266.90	0.079	3.16	.299
*Amb_max*	4	271.01	0.010	24.64	.152
*Amb_max—Sex*	5	271.10	0.010	25.73	.186
*Null*	3	271.83	0.007	37.21	.000
* Sex*	4	272.21	0.006	44.90	.027
* Tal_night*	4	272.61	0.005	54.85	.091
* Tal_night—Sex*	5	272.80	0.004	60.20	.122

All variables demonstrated negative relationships with corticosterone estimates. The negative slope for sex indicates males had lower corticosterone estimates. Site was used as a random effect in all models and the null model included only the random effect.

## 4 Discussion

### 4.1 Laboratory validation

While we agree with other authors regarding the power of biological validation of GC measurements (Sheriff et al., 2011; Touma & Palme, 2005), the limited feasibility of validation must also be acknowledged for some species. The logistical difficulties of a long‐term stress trial in wild animals can lead to mixed results when conducting a biological validation of hair‐based stress analyses (Koren et al., 2002; Mastromonaco et al., 2014). For example, Mastromonaco et al. (2014) were only able to resample 12 of the original 23 eastern chipmunks used in a biological validation of hair samples (ACTH challenge), and only three of the five samples in their experimental group exhibited elevated glucocorticoid levels. Additionally, some species of conservation interest, such as the American pika, would likely exhibit high mortality during a rigorous stress trial (MacArthur & Wang, 1973). However, hair‐based measurements of GC have been validated in other lagomorph species; for example, Peric et al. (2017) documented a significant increase in cortisol incorporated into hair samples from New Zealand white rabbits after stressful events.

Using individual‐based comparisons, we documented a limited connection between plasma‐ and hair‐based estimates and no connection between hair GC and fecal GCM. This lack of correspondence may be attributable to the different time periods over which these sample sources are sensitive. Levels of GC in the bloodstream can be significantly elevated in just a few minutes after a stressful stimulus (Sheriff et al., 2011), and plasma measurements in our study reflect an acute stress response. GCM measurements reflect GC levels on a time scale of several hours or days prior to collection; however, levels of GC in hair represent the accumulation of GC during the relatively long period of hair growth (Koren et al., 2002; Yang, Lan, Yan, Xue, & Dail, 1998). Accordingly, our hair samples likely measured long‐term chronic stress following the entire summer molting period, whereas plasma or fecal samples reflected chronic stress experienced during the few hours or days before the time of capture. Additionally, plasma GC, fecal GCM, and hair GC measure slightly different hormonal signatures, and other lagomorph studies have documented a lack of correlation among alternative stress metrics (Cabezas, Blas, Marchant, & Moreno, 2007; Monclús, Rödel, Palme, Von Holst, & De Miguel, 2006). For example, only free GCs (those not bound to the carrier protein, corticosterone‐binding globulin) circulating in the bloodstream are metabolized by the liver and converted into GCMs; thus, fecal GCM levels have been shown to mirror free GC levels, but not total GC levels in plasma (Sheriff, Krebs, & Boonstra, 2010). The manner in which GCs are incorporated into hair is largely unknown, so questions remain about whether circulating free GC concentration in the blood is proportionately reflected in hair and the influence of confounding factors such as GC contributions from saliva or scents (Sheriff et al., 2011). These temporal and measurement differences are likely responsible for the weak correlations previously observed when hair hormone levels have been compared to those of plasma (Yang et al., 1998) and fecal samples (Mastromonaco et al., 2014).

One of the potential difficulties of using hair is the apparent decrease in extraction efficiency with higher sample masses. Interestingly, this same pattern was reported in fecal samples for both mourning doves (Millspaugh & Washburn, 2004) and California spotted owls (Tempel & Gutierrez, 2004), reinforcing the need to correct for extraction efficiency. Our approach was to establish a nonlinear relationship to account for this influence. This approach may be preferable when the mass or number of samples is low, as it obviates the need to standardize sample sizes by eliminating smaller samples or truncating larger ones. Of course, this nonlinear relationship suggests that estimates based on low sample masses are less precise (another reason not to standardize samples to the lowest sample mass). Of particular note, the nine hair samples used here to contrast with estimates of fecal GCM and plasma GC were generally low in mass, potentially contributing to the weak relationship observed. We agree with Macbeth, Cattet, Stenhouse, Gibeau, and Janz (2010) who recommended a minimum sample weight of 5 g when analyzing GC from hair. In our analysis, the relationship between sample mass and GC estimates was approximately linear for samples larger than 5 g and the residuals of our model explaining extraction efficiency were disproportionally high for the low sample masses. We further recommend researchers consider the influence of sample mass in GC extraction efficiencies even for larger samples; our data suggest such effects continue even at higher sample masses.

To summarize, we recommend researchers employ a traditional validation technique if possible when applying a novel stress analysis protocol. When this is not feasible, as in our case, we suggest a cautious approach to cross‐validation as each sample source may measure unique temporal and physiological elements of stress. Additionally, we emphasize the importance of considering and correcting for the influence of extraction efficiency. Sample mass was demonstrated here and elsewhere to have a strong influence on estimated corticosterone concentrations. We recommend standardizing sample masses, when possible, above 5 mg; however, the relationship between extraction efficiency and sample mass may be species specific, so this cutoff may need to be reassessed, especially in species with hair that is coarser. When logistical considerations make standardizing sample mass impractical, we recommend correcting for extraction efficiency by assessing for a nonlinear correlation between sample mass and GC estimates. The resulting equation can then be used to correct for the influence of sample mass.

### 4.2 Field study

We demonstrated the utility of hair samples by directly investigating factors influencing long‐term chronic stress at the individual level. The sensitivity of this analysis allowed us to evaluate the American pika for several patterns of stress hormone activity well‐documented in other mammals. Our results showed that hair GC was mainly influenced by body size, a pattern perhaps mediated by individual differences in mass‐specific metabolic rates. In mammals, there is a negative relationship between body mass and GC concentration, underpinned by a relative increase in mass‐specific metabolic rate with decreasing body mass (Haase et al., 2016). As the production but not the degradation of GC is a metabolic function, smaller pikas with higher metabolic rates would be more prone to accumulate GC. Furthermore, smaller animals generally lose heat faster due to their higher surface area to volume ratios and would need to elevate their baseline metabolism disproportionally to compensate. Additionally, it may be possible that larger pikas would have longer hair, better insulating them from cold stress or influencing the incorporation of GC into the hair. However, an analysis of New Zealand White rabbits found no influence of hair length or body location on GC estimates using a similar protocol (Comin et al., 2012). To our knowledge, this is the first time that a relationship between GC and body size has been reported within a single species; however, pikas may be exceptional given their relatively high metabolic rate (Lovegrove, 2003), and further investigation is needed to determine whether this pattern is prevalent within other mammals.

Here, we report perhaps the first direct connection between chronic stress and microclimate variation in the American pika, a species with a reputation for narrow thermal tolerance (Moyer‐Horner, Mathewson, Jones, Kearney, & Porter, 2015; Smith, 1974). Our results further support the potential for the negative effects of chronic cold stress in this species (Beever, Ray, Mote, & Wilkening, 2010; Beever et al., 2011; Jeffress et al., 2013; Ray, Beever, & Loarie, 2012; Schwalm et al., 2016). The increase in GC observed at colder sites could be a function of when our hair samples were grown. The American pika molts twice each year, during summer and fall (Smith & Weston, 1990). While we cannot determine the exact time period over which stress was measured, our samples likely captured the GC profile of pikas just after the summer molt, which typically occurs around June to mid‐July (Krear, 1965). An increase in GC associated with lower ambient temperatures may indicate the necessity of a higher metabolic rate to maintain homeostasis in colder conditions (Lovegrove, 2003), particularly during a molt. Being a small alpine mammal that does not hibernate, the American pika may be especially dependent on a fine‐tuned metabolic rate, given that smaller animals would be disproportionately affected by low temperatures (Moyer‐Horner et al., 2015). As a case in point, Boratyński, Jefimow, and Wojciechowski (2016) found that both the basal metabolic rate and nonshivering thermogenesis in Siberian hamsters were highly plastic during the summer months to meet local thermal conditions. If such patterns generalize to the current study, the elevational pattern of GC reported here may represent the metabolic plasticity of pikas to local thermal conditions. We should note that these data do not refute the potential for heat stress in pikas, as the record of GC in our hair samples would likely have been from early summer when the risk of heat stress was minimal. Additionally, it was the mid‐elevational sites that had the lowest stress levels along each of the respective transects, a pattern indicating these sites may have been thermally optimal for pikas, with the potential for stress at lower or higher temperatures.

The lower stress levels reported here for male pikas match the general pattern observed in most mammalian species (Reeder & Kramer, 2005). As both male and female pikas are highly territorial (Smith & Weston, 1990), this aspect of behavior is unlikely to contribute to sex‐specific differences in stress. Our samples likely represented the postbreeding period and thus would not capture the increase in stress associated with mating found in other small mammals (Koren, Mokady, & Geffen, 2008). However, our sampling period coincided with gestation and lactation. The costly metabolic demands of rearing offspring may be responsible for elevated stress hormone levels in female mammals (Gittleman & Thompson, 1988; Wade & Schneider, 1992). Interestingly, female pikas possess a larger adrenal gland than males (Smith & Weston, 1990), potentially to meet these physiological demands. However, the relative stress level of each sex may fluctuate seasonally as males and females perform differing tasks, which could decouple acute and long‐term stress measurements. For instance, Wilkening et al. (2013) reported higher GCM levels in male pikas, but a longer duration of GCM response to an acute stressor in females.

One of the known limitations of the elevational transect experimental design is the high degree of covariation among microclimate variables typically observed, which can preclude the identification of specific climate influences (Sundqvist, Sanders, & Wardle, 2013). The high degree of covariation within our microclimate estimates was indicative of the overarching influence of elevation on climate within our sample area. As such, our measurements likely represent relative microclimate differences between our sites, independent of time period. Fittingly, our direct measurement of *Amb_max* was highly related to mean annual temperature at our sites for 2014 (*R* ^2^ = .878, *F* = 43.2, *df* = 6, *p* < .001) using downscaled weather station data from the climateWNA model (Wang, Hamann, Spittlehouse, & Murdock, 2012). While this addresses our concern over using microclimate measurements taken subsequent to our hair samples and over a short period of time, it does render identifying more specific climate influences challenging with this dataset. This limitation could potentially be addressed by careful selection of additional elevational transects in the future.

In conclusion, we suggest a cautionary approach when attempting GC measurements in a species without the ability to validate the methodology. Identifying biologically relevant and well‐supported relationships such as GC covariance with body size can assist in the development of novel measurement protocols. In addition, cross‐referencing GC metrics among analysis methods may support novel applications in some cases. However, we urge careful consideration of sample type in addressing physiological stress in wildlife, as sample sources vary in the time periods over which they actively measure stress. Finally, we report the only known correlation between directly measured physiological stress and climate variation in the American pika. Our results add to the recent evidence of cold stress in pikas (Beever et al., 2010, 2011; 2011; Ray et al., 2012; Jeffress et al., 2013; Schwalm et al., 2016). We suspect that the elevated metabolic rate needed to endure colder ambient conditions as a small bodied, nonhibernating mammal may be responsible for the elevated GC levels reported here. Further research assessing physiological stress in the American pika may assist in conservation and monitoring efforts as we enter a period of rapid environmental change.

## Conflict of Interest

None declared.
